# Effectiveness of the EMPOWER-PAR Intervention in Improving Clinical Outcomes of Type 2 Diabetes Mellitus in Primary Care: A Pragmatic Cluster Randomised Controlled Trial

**DOI:** 10.1186/s12875-016-0557-1

**Published:** 2016-11-14

**Authors:** Anis Safura Ramli, Sharmini Selvarajah, Maryam Hannah Daud, Jamaiyah Haniff, Suraya Abdul-Razak, Tg Mohd Ikhwan Tg-Abu-Bakar-Sidik, Mohamad Adam Bujang, Boon How Chew, Thuhairah Rahman, Seng Fah Tong, Asrul Akmal Shafie, Verna K. M. Lee, Kien Keat Ng, Farnaza Ariffin, Hasidah Abdul-Hamid, Md Yasin Mazapuspavina, Nafiza Mat-Nasir, Chun W. Chan, Abdul Rahman Yong-Rafidah, Mastura Ismail, Sharmila Lakshmanan, Wilson H. H. Low, for the EMPOWER-PAR Investigators

**Affiliations:** 1 Discipline of Primary Care Medicine, Faculty of Medicine, Universiti Teknologi MARA (UiTM), Selayang Campus, Jalan Prima Selayang 7, 68100 Batu Caves, Selangor Malaysia; 2 Institute for Pathology, Laboratory and Forensic Medicine (I-PPerForM), Universiti Teknologi MARA (UiTM), Sungai Buloh Campus, Jalan Hospital, 47000 Sungai Buloh, Selangor Malaysia; 3 Julius Center for Health Sciences and Primary Care, University Medical Centre Utrecht, Utrecht, Netherlands; 4 Clinical Epidemiology Unit, National Clinical Research Centre, Ministry of Health Malaysia, Kuala Lumpur, Malaysia; 5 Department of Family Medicine, Faculty of Medicine and Health Sciences, Universiti Putra Malaysia, Serdang, Selangor Malaysia; 6 Department of Family Medicine, Faculty of Medicine, Universiti Kebangsaan Malaysia, Kuala Lumpur, Malaysia; 7 School of Pharmaceutical Sciences, Universiti Sains Malaysia, Penang, Malaysia; 8 Department of Family Medicine, School of Medicine, International Medical University, Bukit Jalil, Kuala Lumpur, Malaysia; 9 Faculty of Medicine & Defense Health, National Defense University of Malaysia, Sungai Besi Camp, Kuala Lumpur, Malaysia; 10 Discipline of Family Medicine, Faculty of Medicine, Cyberjaya University College of Medical Sciences, Cyberjaya, Selangor Malaysia; 11 Klinik Kesihatan Seremban 2, Seremban, Negeri Sembilan Malaysia; 12 Monash Health, Monash Medical Centre, Clayton Campus, Clayton, VIC Australia

**Keywords:** Type 2 diabetes mellitus, Chronic disease management, Chronic care model, Multifaceted intervention, Clinical outcomes, Primary care, Family medicine

## Abstract

**Background:**

The chronic care model was proven effective in improving clinical outcomes of diabetes in developed countries. However, evidence in developing countries is scarce. The objective of this study was to evaluate the effectiveness of EMPOWER-PAR intervention (based on the chronic care model) in improving clinical outcomes for type 2 diabetes mellitus using readily available resources in the Malaysian public primary care setting.

**Methods:**

This was a pragmatic, cluster-randomised, parallel, matched pair, controlled trial using participatory action research approach, conducted in 10 public primary care clinics in Malaysia. Five clinics were randomly selected to provide the EMPOWER-PAR intervention for 1 year and another five clinics continued with usual care. Patients who fulfilled the criteria were recruited over a 2-week period by each clinic. The obligatory intervention components were designed based on four elements of the chronic care model i.e. healthcare organisation, delivery system design, self-management support and decision support. The primary outcome was the change in the proportion of patients achieving HbA1c < 6.5%. Secondary outcomes were the change in proportion of patients achieving targets for blood pressure, lipid profile, body mass index and waist circumference. Intention to treat analysis was performed for all outcome measures. A generalised estimating equation method was used to account for baseline differences and clustering effect.

**Results:**

A total of 888 type 2 diabetes mellitus patients were recruited at baseline (intervention: 471 vs. control: 417). At 1-year, 96.6 and 97.8% of patients in the intervention and control groups completed the study, respectively. The baseline demographic and clinical characteristics of both groups were comparable. The change in the proportion of patients achieving HbA1c target was significantly higher in the intervention compared to the control group (intervention: 3.0% vs. control: −4.1%, *P* < 0.002). Patients who received the EMPOWER-PAR intervention were twice more likely to achieve HbA1c target compared to those in the control group (adjusted OR 2.16, 95% CI 1.34–3.50, *P* < 0.002). However, there was no significant improvement found in the secondary outcomes.

**Conclusions:**

This study demonstrates that the EMPOWER-PAR intervention was effective in improving the primary outcome for type 2 diabetes in the Malaysian public primary care setting.

**Trial registration:**

Registered with: ClinicalTrials.gov.: NCT01545401. Date of registration: 1st March 2012.

**Electronic supplementary material:**

The online version of this article (doi:10.1186/s12875-016-0557-1) contains supplementary material, which is available to authorized users.

## Background

It is estimated that 415 million people suffer from type 2 diabetes mellitus (T2DM) with the global prevalence of 8.8% [1]. T2DM is the 7th leading cause of death worldwide [2]. The number is predicted to increase beyond 642 million people within the next 25 years [1] and deaths attributable to T2DM will double by 2030 [2]. Malaysia, a multi-ethnic nation consisting predominantly of Malays, Chinese and Indians, is also experiencing a T2DM epidemic. Prevalence of T2DM among adults aged ≥ 18 years old has dramatically increased from 6.3% in 1986, 8.3% in 1996, and 11.6% in 2006 to an astounding 15.2% in 2011 [3]. It has been projected that Malaysia would have a total of 3.2 million people with T2DM by the year 2030 [1]. T2DM was the 9th leading cause of disease burden in Malaysia as measured by the Disability-Adjusted Life Years (DALYs) [4] and the 6th leading cause of premature death as measured by the number of years of life lost (YLLs) [5].

Majority of T2DM patients in Malaysia are being managed in the public primary care setting as the services are heavily subsidised by the government and patients pay minimal sum for treatment [6]. In the private sector, payments are largely borne by the patients or private medical insurance [7]. Without medical insurance, it is often too expensive for patients with T2DM to receive care in this setting. Therefore, the over-subsidised and resource-constrained public primary care sector is overloaded to provide care to the majority of T2DM patients [6, 7].

Even though Malaysian public primary care providers struggle hard to meet evidence-based standards of care as recommended by the clinical guidelines, many fall short due to the high workload and constraints in terms of staffing and other resources [7]. Widespread implementation of multidisciplinary team management and delivery of self-management support for T2DM are hampered by shortages of trained personnel [7]. Drug availability is still limited, especially the newer and more expensive hypoglycaemic agents [7]. The increasing burden of managing T2DM presents enormous challenges to the public primary care workforce, resulting in sub-optimal management, poor clinical outcomes and high complication rates [7, 8]. Analysis of the National Diabetes Registry (NDR) involving 70,889 adults with T2DM in the Malaysian public primary care setting demonstrated poor glycaemic control with mean HbA1c of 8.3, and 52.6% received sub-optimal management of related cardiovascular (CV) risk factors [8].

Evidence from developed countries has shown that clinical outcomes of T2DM can be improved with multifaceted interventions based on the chronic care model (CCM) [9–12]. This model promotes that better chronic disease outcome is achieved when a well-coordinated, proactive healthcare team interacts productively with empowered and motivated patients [13–15]. The CCM consists of 6 interrelated key elements which include healthcare organisation, delivery system design, clinical information system, patient self-management support, decision support and use of community resources [13–15]. However, evidence on the effectiveness of the CCM in developing countries is still insufficient. To date, there were only a handful of published studies using CCM in this setting. A small before-and-after study of structured diabetes clinics in primary care in the United Arab Emirates showed that the intervention was successful in improving adherence to diabetes guidelines and increased some aspects of satisfaction with diabetes care [16]. However, the intervention did not result in a statistically significant improvement in clinical outcomes [16]. A recent before-and-after study of multifaceted interventions based on the CCM in the Northern Philippines showed significant decrease in HbA1c (median, from 7.7 to 6.9%, *P* < 0.000) and significant improvement in the proportion achieving good glycaemic control among the participants (37.2 to 50.6%, *P* = 0.014) [17]. The CORFIS study is the only published evidence on the effectiveness of CCM in Malaysia [18]. It was conducted in the private primary care setting and showed significant improvement in the proportion of hypertension patients achieving target blood pressure (BP) after 6 months of intervention [18].

Therefore, further research is needed to evaluate the effectiveness of CCM-based intervention among T2DM patients in the Malaysian public primary care setting, where a larger proportion of these patients are receiving care and where limited resources are often stretched thin. Given the constraints in the public primary care setting, successful implementation of the CCM requires pragmatic utilisation of existing health care resources and participatory approach aiming at empowering primary care providers to improve clinical practice [19, 20]. This led to the objective of this study which was to evaluate the effectiveness of the EMPOWER-PAR intervention (multifaceted chronic disease management strategies designed based on the CCM) in improving clinical outcomes for patients with T2DM using existing health care resources in the Malaysian public primary care setting.

## Methods

### Study design

This was a pragmatic, cluster-randomised, parallel, matched pair, controlled trial using participatory action research (PAR) approach [20] in public primary care clinics from two states in Malaysia, which were Wilayah Persekutuan Kuala Lumpur (WPKL) and Selangor (SEL). The pragmatic study design was chosen to maximise external validity to ensure that the results can be generalised to the public primary care system in Malaysia [21]. The study protocol was registered with the clinicaltrial.gov (NCT01545401) and was published in 2014 [22]. This paper reports the findings from the T2DM arm of the study and the reporting is done in accordance with the extension of CONSORT Statements on reporting pragmatic trials and cluster randomised trials [23, 24].

### Site selection and recruitment

All 34 public primary care clinics led by Family Medicine Specialists (FMS) in SEL and WPKL were invited to participate in this study. The FMS were invited to attend a briefing session on the study objectives and methodology. Detailed explanations were given regarding the pragmatic nature of the study design, the eligibility criteria and the concept of PAR approach in implementing the EMPOWER-PAR intervention.

The site feasibility questionnaire (SFQ) was then distributed to all FMS who attended the briefing session. This questionnaire was also sent via email to all FMS who did not attend the session. The SFQ was divided into four sections which included site investigator’s information; clinic location and type, workload and staffing; information on the pre-existing delivery of care for T2DM; and site investigator’s interest in participating in this study. In the ‘pre-existing delivery of care for T2DM’ section of the SFQ, the ‘green book’ referred to the booklets which are widely used in majority of the public primary care clinics in Malaysia. It is made out of two books, an A5-size medical record booklet which is kept by the clinic and a smaller A6-size ‘mini green book’ which is kept by the patient. The ‘green book’ contains information on symptoms, evidence of complications, medications, vital signs and investigations including blood results. The ‘mini green book’ records similar clinical data for follow-up treatment purposes. However, it does not contain CV risk or self-management information.

The SFQ was returned to the investigators after two weeks, either by post or email. The clinics were then assessed for the following eligibility criteria:

1. had ≥ 500 patients with T2DM in the registry.
2. had an FMS who were keen to participate and willing to lead the team.
3. had the capacity and willing to implement the obligatory components of the EMPOWER-PAR intervention.
4. was located within 70 km from the central laboratory as the blood samples were transported back to the centre for analysis.

Out of the 34 sites, only 20 fulfilled the eligibility criteria to enter the study. Finding of the site feasibility assessment is provided in the Additional file 1. These 20 clinics were then matched according to their geographical locations (urban or sub-urban), workload and staffing into 10 pairs. Clinics were matched according to these covariates as they were likely to affect the outcome variables, as the intervention was delivered at the cluster (clinics) level. This was employed to ensure similarity between the intervention and control group.

The investigators used computer generated tables to randomly select five out of the 10 matched-pairs to be included into the study. Then, one clinic in each pair was randomly allocated into the intervention or control arms.

### Patient recruitment

Consecutive T2DM patients who attended the clinics within the 2-week recruitment period were given the patient information sheet and interviewed by the investigators in the waiting area. Screening was conducted to identify eligible participants based on the inclusion and exclusion criteria. Eligible patients were then invited to participate and informed consents were obtained from those who were willing to participate.

### Inclusion criteria

Males and females aged ≥ 18 years who:

1. were diagnosed with T2DM, or on treatment for T2DM
2. and received follow-up care for T2DM in the same clinic at least once in the last 1 year

### Exclusion criteria

1. type 1 diabetes mellitus
2. receiving renal dialysis
3. presented with severe hypertension (HPT) (Systolic BP > 180 mmHg and/or Diastolic BP > 110 mmHg) at recruitment
4. diagnosed with conditions resulting in secondary hypertension
5. diagnosed with circulatory disorders requiring referral to secondary care over the last one year (e.g. unstable angina, heart attack, stroke, transient ischaemic attacks)
6. receiving shared care at primary and secondary care centres for complications of T2DM
7. pregnant
8. enrolled in another study

During the 1-year intervention period, all patients in the intervention arm were required to be seen at least twice by the Chronic Disease Management (CDM) team from each clinic. Patients who did not comply with the follow-up requirement were considered as lost to follow-up. During the course of the study, there was no limit to the number of clinic visits that a patient was allowed to make in either the intervention or control groups.

### The EMPOWER-PAR intervention

The EMPOWER-PAR intervention was designed based on the six interrelated elements of the CCM. The details of its development have been described in the study protocol [22]. It consisted of three obligatory components and two optional components utilising readily available and existing resources in the Malaysian public primary care setting. The aim was to have a productive interaction between the empowered CDM team and the informed, empowered T2DM patients [22]. Table 1 summarised the components of the EMPOWER-PAR intervention according to their respective CCM elements.

**Table 1:** The obligatory and the optional components of the EMPOWER-PAR intervention and the related CCM elements

CCM elements	Obligatory EMPOWER-PAR Intervention	Target level
• Organisation of Health Care • Delivery System Design	Creating/Strengthening a CDM Team-a multidisciplinary team led by the FMS to improve coordination of care for T2DM and co-existing CV risk factors	Primary Care Providers
• Decision Support	Utilising the national Clinical Practice Guidelines (CPG) for T2DM to aid management and prescribing	Primary Care Providers
• Self-Management Support	Utilising the Global CV Risks Self-Management Booklet to support patients self-management	T2DM Patients
CCM Elements	Optional EMPOWER-PAR Intervention	Target Level
• Clinical Information System	Utilising clinical information system and conducting clinical audits to track progress through reporting outcomes to patients and providers	Primary Care Providers
• Community Resources and Policy	Utilising community resources to support and sustain care	Primary Care Providers

The EMPOWER-PAR intervention was unique as it was designed based on the entire CCM elements using readily available resources. Although there was robust evidence supporting the individual elements of the CCM, there is still paucity in the literature regarding implementation of the entire CCM as a multifaceted intervention, especially in a resource-constrained primary care setting. With the exception of several studies [16–18], previous studies implementing the CCM elements as multifaceted interventions have been conducted in developed countries [9–12]. Similar to CORFIS [18], the EMPOWER-PAR was not designed to differentiate the effectiveness of individual CCM elements in its multifaceted intervention.

### Implementation process of the intervention

**Fig. 1:**
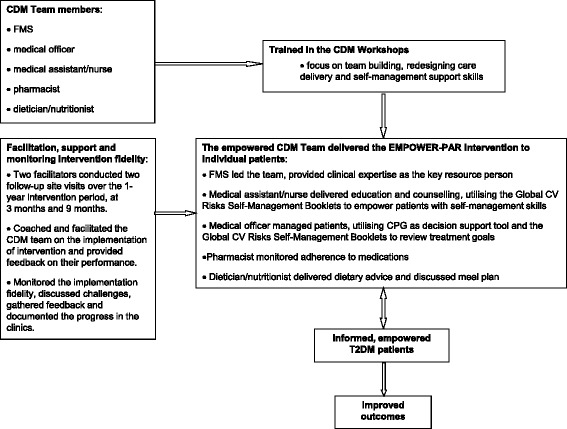
Delivery Structure of the EMPOWER-PAR Intervention

The EMPOWER-PAR intervention was delivered for a period of 1-year. The intervention clinics received the EMPOWER-PAR intervention package, which consisted of CDM Workshops, intervention tools, facilitation and support. The process evaluation of this complex intervention was conducted in accordance with the United Kingdom Medical Research Council guidance [25]. Figure 1 summarised the delivery structure of the EMPOWER-PAR intervention.

The implementation process was conducted in 3 phases as below:

#### Phase 1: Formation and training of the CDM Team

Each intervention clinic identified five CDM Team members who were then trained in the CDM Workshops. Details of the CDM Workshops development, objectives and content were already published in the protocol paper [22]. During the workshop, the CDM Team was trained on team building and to define their roles and responsibilities. They were also trained on how to empower their T2DM patients with knowledge and skills to self-manage their condition using the Global CV Risks Self-Management Booklet as a tool. This included improving the skills of the CDM Team to provide accurate information to their patients regarding the nature of the disease, possible complications, treatment goals and the importance of taking their medications appropriately. Emphasis was given on how to improve provider-patient communication, which has been shown to enhance patient self-management behaviours over time [26].

The PAR approach [20] was applied in implementing the EMPOWER-PAR interventions to ensure that the CDM Team were empowered to make the choice of actions within their constraints to improve their patients’ health outcomes. Each clinic had unique challenges which include shortages or high turnover of medical staff and allied health personnel, high patient load, limited clinic space and time constraints. These clinics also had existing chronic disease care system. Therefore it was impractical and inappropriate to apply a rigid intervention program [22]. With this in mind, the CDM Team from each clinic prepared a proposed intervention plan at the end of the workshop series, which considered their unique constraints. The proposed plan described the roles and responsibilities of each team member, the methods to implement the three obligatory intervention components, and also the steps needed to achieve their goals and ways to overcome their barriers. The planning to implement the optional components was also made by the clinics which had adequate resources. The process of PAR gave the autonomy to the health care providers to determine how best to improve the quality of their patient care [20].

#### Phase 2: Distribution and utilisation of the intervention tools

The CDM Team was expected to utilise the Malaysian CPG and the Quick References (QR) on the Management of T2DM [27] to support their clinical decision making during consultations, and the Global CV Risks Self-Management Booklet to support patients’ empowerment and self-management. This booklet was designed as an educational resource material for patients to understand their conditions, risk factors, potential complications, control targets and how to self-manage their conditions. Details on the development and content of this booklet were already published in the protocol paper [22]. Patients were expected to bring this booklet during their follow-up appointments and the CDM Team members were expected to utilise this booklet to review their progress and empower them with self-management skills. This booklet differs from the traditional ‘mini green book’ which serves as communication tool between doctors. The ‘mini green book’ was not designed as an educational resource material for patients and therefore, contains clinical data which may not be readily understood by them.

#### Phase 3: Facilitation and support to implement the intervention

The intervention clinics received facilitation and support throughout the study period to implement the intervention. An intervention review workshop was also conducted 6 months after the commencement of intervention to allow interactions among the participating clinics and solve any arising problems. CPG training and feedback with regards to their baseline clinical outcomes were also delivered during this workshop [22].

### Monitoring the implementation fidelity of the intervention

Monitoring the implementation fidelity is an essential part of the process evaluation of a complex intervention [25, 28]. In this study, the facilitators monitored the implementation fidelity of the intervention in each clinic to ensure that it was delivered as intended throughout the 1-year period. Data on implementation fidelity was collected by the facilitators through observation during the site visits. Fidelity monitoring was focused on the implementation of obligatory components of the EMPOWER-PAR intervention. Observation data was captured in writing by the facilitators using a standardised report form, which was later compiled by the chief facilitator. Feedback was also gathered from the CDM Team with regards to their barriers and challenges in implementing the intervention. Facilitators also gave feedback to the intervention clinics with regards to their performance. Meetings amongst the facilitators were conducted at least three times over the 1-year study period to discuss the implementation fidelity in each clinic. Variations in the implementation fidelity between each clinic were minimised through these strategies of facilitation, support and close monitoring.

### The control

The control clinics continued with usual care with no additional intervention during the 1-year period. Allied health personnel were available in the control clinics but they may not be functioning as a team in managing T2DM. The control clinics have access to CPG as these are readily available resources. However, they did not receive CPG training and CPG utilisation was not emphasised or monitored. The CDM workshop modules and intervention tools were made available to the control clinics at the end of the study. There was no other additional resource allocated to either the intervention or the control group.

### Outcome measures

Outcome measures were obtained from both intervention and control clinics at baseline and one year after the commencement of the intervention. The target values for the primary and secondary outcome measures were based on the national CPG for T2DM [27]. Definition of the outcome categories at 1-year follow-up is summarised in the Additional file 2.

### Primary outcome

Primary outcome was measured by the change in the proportion of patients achieving glycaemic target of HbA1c < 6.5% (48 mmol/mol).

### Secondary outcome

Secondary outcomes were measured by changes in the proportions of patients achieving the following targets:

- BP ≤ 130/80 mmHg
- BMI < 23 kg/m^2^
- Waist Circumference (WC) < 90 cm for men, < 80 cm for women
- Total cholesterol (TC) ≤ 4.5 mmol/L
- Triglycerides (TG) ≤ 1.7 mmol/L
- Low density lipoprotein cholesterol (LDL-c) ≤ 2.6 mmol/L
- High density lipoprotein cholesterol (HDL-c) ≥ 1.1 mmol/L

### Data collection and study procedures

Data were obtained from both the intervention and control clinics at baseline and at 1-year follow-up. Baseline data were collected in June 2012 – December 2012, the intervention was delivered in January 2013 – December 2013 and outcome data were collected in January 2014 – June 2014.

All interviewers and investigators were trained regarding the study procedures prior to the conduct of the study to minimise variability in the method of data collection. At baseline, an interview and physical examinations were conducted. Fasting venous blood samples were obtained. Clinically important events such as hypoglycaemia, drug-related adverse events, hospitalisation or deaths were recorded throughout the study period. Details on the demographic and anthropometric data collection procedures were already described in the protocol paper [22].

### Blood sampling and biochemistry profile

The baseline and outcome blood samples were analysed at the Centre for Pathology and Diagnostic Research Laboratory (CPDRL), Universiti Teknologi MARA (UiTM) which is an ISO 15189:2007 accredited laboratory (SAMM 688). Details of the blood sampling and laboratory analysis were already described in the protocol paper [22].

### Sample size calculation

The sample size was calculated using the randomised clustered trial design with PASS software (Copyright (c) 2009 by Dr Jerry L. Hintze, All Rights Reserved). Based on the results reported in previous studies [10, 12], the intervention was expected to detect 25% change in the proportion of subjects achieving target HbA1c < 6.5% from baseline and between the intervention and control groups. As this was a randomised cluster study, the ‘design effect’ was taken into account during sample size calculation. The intra-cluster correlation coefficients (ICC) in cluster primary care trials were generally lower than ρ = 0.05 [29]. If m is the cluster size (assumed to be the same for all clusters), then the inflation factor, or ‘design effect’ associated with cluster randomisation is 1 + (m − 1)ρ [24]. Therefore, for a cluster of 10, the ICC was translated into a design effect of 1.5. Considering this value, a sample size of 626 (313 in each arm) was obtained by sampling 10 clusters (5 intervention vs. 5 control) with 63 subjects from each cluster to detect 25% change in the proportion of subjects achieving target HbA1c < 6.5% from baseline and between treatment groups, with 91% power at 5% significance level. The test statistic used was the two-sided Z-test (unpooled). After allowing for 25% dropout rate, this study aimed to recruit a total sample of 836 T2DM patients at baseline (i.e. 418 in each arm and 84 from each clinic).

### Statistical analyses

Intention to treat analysis was performed for both primary and secondary outcome measures. Missing variables were reviewed and determined if they were missing at random. Multiple imputation was performed using five imputed datasets for the missing variables at follow-up: HbA1c (2.8% missing), systolic BP, diastolic BP, BMI, TC, TG, LDL-c (6% missing) and HDL-c (4.2% missing).

Continuous variables were summarised using means and standard errors, while categorical variables were summarised using counts and percentages. A generalised estimating equation (GEE) method was used to account for randomisation by practices (clustering) for all analyses. No other variable was added to adjust for clustering as stratification and matching of the practices was done prior to randomisation to maximise the balance of covariates between treatment groups. An independent working model was used. Pooled treatment effects for continuous variables were obtained using estimated marginal means.

Cut-off values for definition of outcome categories are provided in the Additional file 2. Comparisons between treatment groups for clinical outcome measures at follow-up were adjusted for baseline values of the outcome measures as well as the cluster effect [30]. The baseline value of a clinical measurement is likely to be the strongest predictor for its follow-up measurement [31]. This adjustment was not determined a priori. Comparisons of outcome measures between treatment groups for changes from baseline were adjusted for cluster effect only. For all analyses, *P* values of less than 0.05 were considered statistically significant. Analyses were performed using IBM SPSS Statistics for Windows, Version 20.0 (IBM Corp., Armonk, NY, USA) and Stata Statistical Software : Release 13.0 (College Station, TX: Stata Corporation LP).

## Results

### Description of the site and population sample

Characteristics of the selected EMPOWER-PAR intervention and control clinics are summarised in Table 2. Distributions of clinics in terms of geographical locations, workload and staffing were similar in both arms.

**Table 2:** Random selection of the eligible clinics and random allocation of the selected clinics into intervention and control arms, *n* = 20

Pair	Geographic Location	Workload (average number of patients seen in the clinic per day)	Staffing (number of doctors and allied health personnel)	1st Stage: Random Selection	2nd Stage: Random Allocation
Pair no. 1	Urban	900	30	√	Intervention
	Urban	900	28		Control
Pair no. 2	Urban	600	27	√	Intervention
	Urban	650	29		Control
Pair no. 3	Urban	330	28	×	
	Sub-urban	350	27		
Pair no. 4	Urban	550	32	√	Intervention
	Urban	500	33		Control
Pair no. 5	Urban	500	50	×	
	Urban	500	51		
Pair no. 6	Sub-urban	300	36	×	
	Urban	200	33		
Pair no. 7	Urban	700	73	×	
	Urban	1000	119		
Pair no. 8	Sub-urban	500	22	√	Intervention
	Sub-urban	500	20		Control
Pair no. 9	Urban	400	44	×	
	Sub-urban	326	41		
Pair no. 10	Sub-urban	350	21	√	Intervention
	Sub-urban	400	19		Control

**Fig. 2:**
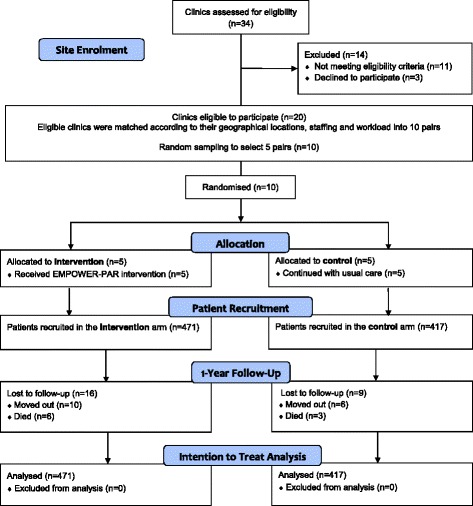
The EMPOWER-PAR CONSORT Flow Diagram

A total of 888 T2DM patients were recruited at baseline; 471 were in the intervention and 417 were in the control group. At 1-year, 455 (96.6%) and 408 (97.8%) patients in the intervention and control groups completed the study, respectively. In the intervention group, 16 (3.4%) patients were lost to follow; 10 patients moved out from the area and 6 deaths were reported. The causes of death were recorded as heart attack (3 patients), cardiac arrest due to heart failure (1 patient), stroke (1 patient) and hyperosmolar hyperglycaemic state (1 patient). In the control group, 9 (2.2%) patients were lost to follow-up; 6 patients moved out and 3 deaths were reported. The causes of death were recorded as heart attack (1 patient), stroke (1 patient) and dyspnoea (1 patient). There was no other clinically important event such as hypoglycaemia or drug-related adverse event reported throughout the study period in both groups. Figure 2 shows the The EMPOWER-PAR CONSORT Flow Diagram [24].

Table 3 shows the baseline sociodemographic and clinical characteristics of the participants. The two groups were comparable in terms of age, gender distribution, ethnicity, education attainment, smoking status, coexisting hypertension, history of cardiovascular events (myocardial infarction, stroke and peripheral vascular disease), duration of T2DM and duration of hypertension. However, the proportion of T2DM patients with coexisting hyperlipidaemia was significantly lower in the intervention compared to control group (intervention: 46.9% vs. control: 55.9%, *P* = 0.01). Patients in the intervention group also had a significantly shorter duration of hyperlipidaemia compared to the control group (intervention: 1.8 years, SE ± 0.15 vs. control: 2.6 years, SE ± 0.21, *P* = 0.001). The mean biochemical characteristics of the two groups were also comparable at baseline, except for BMI and HDL-c. The proportions of patients achieving biochemical targets were also comparable at baseline, except for TG.

**Table 3:** Baseline sociodemographic and clinical characteristics of T2DM patients allocated to the intervention and control groups, *n* = 888

Characteristics		Intervention *n* = 471	Control *n* = 417	*P* value
Age, years; Mean (SE)		58 (0.48)	57 (0.5)	0.36
Gender; n (%)				
Males		180 (38.2)	149 (35.7)	0.44
Females		291 (61.8)	268 (64.3)	
Ethnicity; *n* (%)				
Malays		242 (51.4)	190 (45.6)	0.26
Chinese		71 (15.1)	90 (21.6)	
Indians		157 (33.3)	130 (31.2)	
Others		1 (0.2)	7 (1.6)	
Education attainment; *n* (%)				
No education		50 (10.6)	45 (10.8)	0.84
Primary		187 (39.7)	157 (37.6)	
Secondary		197 (41.8)	192 (46.1)	
Tertiary		37 (7.9)	23 (5.5)	
Smoking status; *n* (%)				
Non-smoker		363 (77.1)	330 (79.1)	0.42
Current smoker		66 (14.0)	50 (12.0)	
Ex-smoker		42 (8.9)	37 (8.9)	
Comorbidity; *n* (%)				
Hypertension		349 (74.1)	329 (78.9)	0.09
Hyperlipidaemia		221 (46.9)	233 (55.9)	**0.01**
History of myocardial infarction, stroke or peripheral vascular disease		20 (4.2)	16 (3.8)	0.76
Duration of Medical Conditions, years; Mean (SE)				
Duration of diabetes mellitus		6.5 (0.28)	6.8 (0.29)	0.41
Duration of hypertension		5.5 (0.32)	5.4 (0.32)	0.72
Duration of hyperlipidaemia		1.8 (0.15)	2.6 (0.21)	**0.001**
Biochemical characteristics at baseline; mean (SE)				
HbA1c	(%)	8.4 (0.09)	8.4 (0.09)	0.91
	(mmol/mol)^a^	68.3	68.3	
Systolic BP (mmHg)		139 (0.83)	138 (0.81)	0.60
Diastolic BP (mmHg)		80 (0.42)	80 (0.44)	0.49
BMI (kg/m^2^)		27.6 (0.23)	28.5 (0.29)	**0.01**
WC (cm)		95 (0.47)	96 (0.56)	0.19
TC (mmol/L)		5.3 (0.06)	5.3 (0.05)	0.65
TG (mmol/L)		2.2 (0.07)	2 (0.06)	0.09
LDL-c (mmol/L)		3.2 (0.05)	3.2 (0.05)	0.90
HDL-c (mmol/L)		1.1 (0.01)	1.2 (0.02)	**0.01**
Proportion achieving biochemical targets at baseline; %				
HbA1c < 6.5%/< 48 mmol/mol		15.3	17.0	0.48
BP ≤130/80 mmHg		24.8	25.9	0.72
BMI < 23 kg/m^2^		15.7	12.7	0.20
WC	<90 cm (Men)	11.3	12.5	0.58
	<80 cm (Women)			
TC ≤ 4.5 mmol/L		26.8	26.9	0.97
TG ≤ 1.7 mmol/L		45.4	52.8	**0.03**
LDL-c ≤ 2.6 mmol/L		31.9	31.2	0.82
HDL-c ≥ 1.1 mmol/L		60.9	66.7	0.08

^a^HbA1c in mmol/mol = [10.93 × HbA1c in %] – 23.5

Bold data represents statistically significant results i.e *P* value < 0.05

### Evaluation of the implementation fidelity

Table 4 summarises the pre-existing system of T2DM care in the intervention clinics, changes made during the intervention period and implementation fidelity as observed by the facilitators. Two of the clinics had pre-existing dedicated chronic disease clinic, while three clinics managed their chronic cases together with acute cases in the general outpatient clinic. Those clinics which already had a pre-existing team strengthened their CDM Team through the EMPOWER-PAR intervention, while the clinics without any pre-existing team indentified new members to be trained. Some of the clinics lost their team members during the study period as they were transferred to other clinics out of the region. However, new members were promptly identified and retrained. The CDM delivery system was also reviewed and strengthened in all of the intervention clinics. With regards to T2DM CPG utilisation prior to the intervention period, CPG was available in the FMS room in most clinics. During the intervention period, the facilitators observed that CPG QR was made available in each consultation room and was utilised by team members for decision making process during consultations. With regards to self-management tool, patients carried the ‘mini green book’ prior to the intervention period. During the intervention period, the clinics distributed the Global CV Risks Self-Management Booklet (which was also known as the ‘red book’) to all T2DM patients in their clinics. Utilisation of the ‘red book’ by the CDM Team to support patients’ self-management was also observed by the facilitators. In most clinics, patients kept both the ‘red book’ and the ‘green book’ during the study period. With regards to the implementation of the optional components, most of the clinics continued with the pre-existing system of medical record keeping. Two of the clinics were utilising the community resources through their clinic advisory panel and continued to do so during the intervention period. All intervention clinics were also able to optimally adhere to the methods of implementation which was proposed during the CDM Workshops. Through the process of PAR, the FMS who led the CDM Team in each clinic ensured that the intervention was delivered as intended. A close working relationship was also developed between the facilitators and the CDM Team in each clinic.

**Table 4:** Implementation fidelity of the EMPOWER-PAR intervention

Intervention clinics		Obligatory EMPOWER PAR intervention			Optional EMPOWER PAR intervention	
		Creating/Strengthening a CDM team & CDM delivery system	Utilising T2DM CPG	Utilising the Global CV risks self-management booklet	Utilising clinical information system and conducting clinical audits	Utilising community resources
Clinic 1	Pre-existing system	Pre-existing dedicated chronic disease clinic for T2DM & HPT (appointment system, flow of patients, defaulter tracing etc.) 1 medical officer, 2 nurses, 1 pharmacist and 2 attendants were running this clinic.	CPG was available in the FMS room.	Patients carried the ‘mini green book’.	The clinic utilised the ‘green book’ for medical record keeping. Participated in the National Diabetes Registry program –a national audit for T2DM.	No community involvement.
	Changes made & implementation fidelity	Five existing members were trained in the CDM Workshops, led by the FMS. The CDM Team and the delivery system were further strengthened.	CPG QR was made available in hard and soft copies in each consultation room and was utilised by team members for decision making.	The clinic fully utilised the Global CV Risk Self Management Booklet. The book became popular amongst patients and was coined as the “Power” book.	Continued with the pre-existing system.	Attempts were made but there was no formalised community involvement.
Clinic 2	Pre-existing system	No pre-existing dedicated chronic disease clinic. Acute and chronic cases were seen in the integrated general outpatient clinic. A medical officer and a nurse were in-charge of T2DM patients.	CPG was available in the FMS room.	Patients carried the ‘mini green book’.	The clinic utilised the ‘green book’ for medical record keeping. Participated in the National Diabetes Registry program – a national audit for T2DM.	The clinic had an advisory panel consisted of community members. Participated in the Non-Communicable Disease-1Malaysia (NCD-1 M) programme.
	Changes made & implementation fidelity	Five CDM Team members identified and trained (medical officer, nurse, medical assistant, dietician, and pharmacist, led by the FMS). Two members left at 6-month post intervention, and two new members retrained. The clinic created a new CDM delivery system (appointment system, flow of patients, defaulter tracing etc.)	CPG QR was made available in each consultation room and was utilised by team members for decision making during consultation.	The clinic distributed and utilised the Global CV Risk Self Management Booklet to support patients’ self-management during consultation.	Continued with the pre-existing system.	Continued with the pre-existing system.
Clinic 3	Pre-existing system	No pre-existing dedicated chronic disease clinic. Acute and chronic cases were seen in the integrated general outpatient clinic. A medical officer and a nurse were in-charge of T2DM patients.	CPG was available in each consultation room; however, there was no regular discussion among team member regarding case management according to CPG.	Patients carried the ‘mini green book’.	The clinic utilised the ‘green book’ for medical record keeping. Participated in the National Diabetes Registry program – a national audit for T2DM.	The clinic had an advisory panel consisted of community members. Participated in the NCD-1 M programme.
	Changes made & implementation fidelity	Five CDM Team members identified and trained (medical officer, nurse, medical assistant, dietician, and pharmacist, led by the FMS). The clinic created a new CDM delivery system (appointment system, flow of patients, defaulter tracing etc.)	CPG QR was made available in hard and soft copies in each consultation room and was utilised by team members for decision making. Discussion on case management according to the CPG was done 2-monthly with the FMS.	The clinic distributed and utilised the Global CV Risk Self Management Booklet to support patients’ self-management during consultation. Scheduled patient education series were conducted, which included diabetes conversation maps and cooking demonstration.	Continued with the pre-existing system.	Continued with the pre-existing system.
Clinic 4	Pre-existing system	Pre-existing dedicated chronic disease clinic ran by a team of 7 health care providers.	CPG was available in each consultation room; with online information on management of T2DM.	Patients carried the ‘mini green book’.	The clinic utilised the ‘green book’ for medical record keeping. Participated in the National Diabetes Registry program – a national audit for T2DM.	None.
	Changes made & implementation fidelity	Five existing members were trained in the CDM Workshops, led by the FMS. The CDM Team and the delivery system were further strengthened through team building and cooperation.	CPG QR utilisation was further strengthened in decision-making process during consultation.	The clinic distributed and utilised the Global CV Risk Self Management Booklet to support patients’ self-management during consultation. Formation of structured diabetes education program and Medication and Therapeutic Adherence Counselling (MTAC).	Continued with the pre-existing system.	Not developed.
Clinic 5	Pre-existing system	No pre-existing dedicated chronic disease clinic. Acute and chronic cases were seen in the integrated general outpatient clinic. One staff nurse handled T2DM cases.	CPG was not available at the nurses’ counter or in the doctors’ consultation rooms	Patients carried the ‘mini green book’.	The clinic has its own diabetes registry, prepared and updated by the AMO regularly. AMO was familiar with SPSS and utilised it to analyse patients’ data.	This is a new clinic in a new modern township, consisting of young working families. There was no engagement with the community resources.
	Changes made & implementation fidelity	Five CDM Team members were identified and trained. FMS was transferred out; a staff nurse took over the leadership of the team. Two medical officers were assigned to see patients with chronic diseases in the morning every day.	CPG QR was made available in the consultation rooms and the nurses’ counter, and was utilised by team members for decision making.	The clinic distributed and utilised the Global CV Risk Self Management Booklet to support patients’ self-management during consultation. Some patients found it useful, but some forgot to bring along during follow-up appointments.	The clinic utilised their registry for clinical audit and tracing defaulters.	Not developed.

### Results by outcome

Table 5 shows the mean change in clinical outcomes at 1-year follow-up. The intervention group showed significant reduction in the mean HbA1c compared to control, which showed an increase in the mean HbA1c (intervention: −0.1%, SE ± 0.06 vs. control: 0.2% SE ± 0.09, *P* = 0.003). For diastolic BP, although both groups showed an increment at 1-year follow-up, the intervention group had a significantly lower mean change in diastolic BP compared to the control group (intervention: 0.4 mmHg, SE ± 0.43 vs. control: 1.9 mmHg SE ± 0.47, *P* = 0.02).

**Table 5:** Mean change in clinical outcomes of T2DM patients at 1-year follow-up

Clinical outcomes		Intervention			Control			Model summary^b^
		Baseline mean (SE)	Follow-up mean (SE)	Change^a^ (SE)	Baseline mean (SE)	Follow-up mean (SE)	Change^a^ (SE)	*P* value^c^
HbA1c	(%)	8.4 (0.09)	8.3 (0.09)	−0.1 (0.06)	8.4 (0.09)	8.5 (0.1)	0.2 (0.07)	**0.003**
	(mmol/mol)^d^	68.3	67.2	−22.4	68.3	69.4	−21.3	
Systolic BP (mmHg)		139 (0.83)	139 (0.86)	−0.3 (0.78)	138 (0.81)	140 (0.92)	1.7 (0.75)	0.08
Diastolic BP (mmHg)		80 (0.42)	81 (0.44)	0.4 (0.43)	80 (0.44)	82 (0.5)	1.9 (0.47)	**0.02**
BMI (kg/m^2^)		27.6 (0.23)	27.8 (0.23)	0.2 (0.08)	28.5 (0.29)	28.6 (0.27)	0.1 (0.14)	0.64
WC (cm)		95 (0.47)	97 (0.56)	2 (0.33)	96 (0.56)	97 (0.64)	1.2 (0.37)	0.08
TC (mmol/L)		5.3 (0.06)	5.2 (0.05)	−0.1 (0.05)	5.3 (0.05)	5.2 (0.05)	−0.1 (0.05)	0.90
TG (mmol/L)		2.2 (0.07)	2.1 (0.05)	−0.1 (0.06)	2 (0.06)	2 (0.05)	−0.1 (0.05)	0.64
LDL-c ≤ 2.6 mmol/L		3.2 (0.05)	3.1 (0.05)	−0.02 (0.04)	3.2 (0.05)	3.1 (0.04)	−0.03 (0.04)	0.84
HDL-c ≥ 1.1 mmol/L		1.1 (0.01)	1.2 (0.01)	0.02 (0.01)	1.2 (0.02)	1.3 (0.02)	0.05 (0.02)	0.09

Intention to treat analysis was performed to determine the mean change in clinical outcome measures

^a^Change from baseline (standard error) unadjusted

^b^Mean change from baseline compared between treatment groups, adjusted for cluster effect using GEE

^c^Significance of intervention term in model

^d^HbA1c in mmol/mol = [10.93 × HbA1c in %] – 23.5

Bold data represents statistically significant results i.e *P* value < 0.05

Table 6 shows the distributions of patients according to the outcome categories at 1-year follow-up. For HbA1c, the proportion of patients in the ‘improving’ category was higher in the intervention group (7.3%) compared to the control group (3.2%), while the proportion of patients in the ‘deteriorating’ category was lower in the intervention group (4.2%) compared to the control group (7.3%), and this trend was significant (*P* = 0.004). There was no significant trend observed in the secondary outcome measures.

**Table 6:** Distribution of T2DM patients according to the outcome categories at 1-year follow-up

Outcome Categories	Group	Deteriorating	Poor, no change	Good, no change	Improving	*P* value
		*n* (%)	*n* (%)	*n* (%)	*n* (%)	
Primary outcome						
HbA1c	Intervention	20 (4.2)	365 (77.4)	52 (11)	34 (7.3)	**0.004**
	Control	31 (7.3)	333 (79.8)	40 (9.7)	13 (3.2)	
Secondary outcome						
BP	Intervention	58 (12.2)	298 (63.4)	59 (12.6)	56 (11.8)	0.15
	Control	61 (14.6)	268 (64.4)	47 (11.3)	41 (9.7)	
BMI	Intervention	18 (3.9)	380 (80.8)	56 (11.8)	17 (3.5)	0.37
	Control	10 (2.5)	357 (85.6)	43 (10.2)	7 (1.7)	
WC	Intervention	16 (4.8)	286 (86.4)	17 (5.1)	12 (3.6)	0.72
	Control	15 (4.5)	285 (85.8)	24 (7.2)	8 (2.4)	
TC	Intervention	48 (10.1)	284 (60.2)	78 (16.6)	61 (13)	0.93
	Control	40 (9.6)	255 (61.2)	72 (17.2)	50 (12)	
TG	Intervention	65 (13.8)	185 (39.3)	149 (31.6)	72 (15.2)	0.32
	Control	52 (12.5)	144 (34.6)	168 (40.2)	53 (12.6)	
LDL-c	Intervention	56 (12.4)	249 (54.8)	89 (19.5)	61 (13.3)	0.45
	Control	50 (12.7)	228 (57.3)	74 (18.5)	46 (11.5)	
HDL-c	Intervention	44 (9.4)	134 (28.4)	243 (51.5)	50 (10.7)	0.11
	Control	34 (8.1)	96 (23)	244 (58.6)	43 (10.4)	

Intention to treat analysis was performed for primary and secondary outcome measures

Bold data represents statistically significant results i.e *P* value < 0.05

Table **7** shows the effectiveness of the EMPOWER-PAR intervention and the changes in the proportion of patients achieving primary and secondary outcome measures at 1-year follow-up. The change in the proportion of patients achieving HbA1c target was significantly higher in the intervention group compared to the control group (intervention: 3.0% vs. control: −4.1%, *P* < 0.002). There was no significant difference in the change of the proportion of patients achieving target in all of the secondary outcome measures between the intervention and control groups. Patients who received the EMPOWER-PAR intervention were twice more likely to achieve HbA1c target compared to those in the control group (adjusted OR 2.16, 95% CI 1.34–3.50, *P* < 0.002). However, there was no significant difference found between the two groups in all of the secondary outcome measures (BP, BMI, WC, TC, TG, LDL-c and HDL-c).

**Table 7:** Effectiveness of the EMPOWER-PAR intervention in achieving the primary and secondary outcome measures at 1-year follow-up

Clinical outcome measures		Intervention			Control			Model summary^b^	
		Baseline %	Follow-up %	Change^a^ %	Baseline %	Follow-up %	Change^a^ %	Odds Ratio (95% CI)^c^	*P* value^d^
Primary outcome									
HbA1c	<6.5%/	15.3	18.3	3.0	17.0	12.9	−4.1	2.16 (1.34, 3.50)	**0.002**
	<48 mmol/mol								
Secondary outcomes									
BP ≤ 130/80 mmHg		24.8	24.4	−0.4	25.9	21.1	−4.8	1.27 (0.91, 1.78)	0.16
BMI < 22.9 kg/m2		15.7	15.4	−0.3	12.7	11.9	−0.8	1.27 (0.70, 2.31)	0.44
WC	<90 cm (M)	11.3	8.8	−2.5	12.5	9.6	−2.9	1.01 (0.53, 1.93)	0.97
	<80 cm (F)								
TC ≤ 4.5 mmol/L		26.8	29.6	2.8	26.9	29.2	2.3	1.03 (0.74, 1.43)	0.86
TG ≤ 1.7 mmol/L		45.4	46.9	1.5	52.8	52.8	0	0.87 (0.65, 1.18)	0.38
LDL-c ≤ 2.6 mmol/L		31.9	32.5	0.6	31.2	29.8	−1.4	1.15 (0.83, 1.60)	0.41
HDL-c ≥ 1.1 mmol/L		60.9	62.2	1.3	66.7	69.0	2.3	0.79 (0.56, 1.12)	0.19

Intention to treat analysis was performed for primary and secondary outcome measures

^a^Change in the proportion of patients achieving clinical outcomes: Follow-up - Baseline

^b^Estimates were derived using GEE. Results were adjusted for baseline values and cluster effect

^c^Odds for achieving clinical outcome measures in the intervention group compared with control group

^d^Significance of intervention term in model

Bold data represents statistically significant results i.e *P* value < 0.05

Results of the other outcome measures as stipulated in the study protocol [22] will be reported in separate papers. These include the process of care for T2DM management, prescribing patterns, medication adherence level, patients’ assessment of the chronic illness care, qualitative analysis of health care providers’ perceptions, attitudes, experiences and perceived barriers in implementing the intervention and cost-effectiveness analysis of the EMPOWER-PAR intervention.

## Discussions

### The CDM system change

The EMPOWER-PAR was one of the first pragmatic randomised controlled trials of multifaceted interventions based on the CCM conducted in a resource-constrained public primary care setting in a developing country. This study shows that the clinics receiving the EMPOWER-PAR intervention package were capable of strengthening their CDM system by implementing the obligatory intervention components. These included strengthening the roles of primary care providers in the CDM team, reinforcing their adherence to T2DM CPG to support evidence based decision making, and enhancing their skills to improve patients’ self-management behaviours. These components were designed based on the four CCM elements, namely healthcare organisation, delivery system design, decision support and self-management support. Interventions involving delivery system design reported the largest improvements in patient outcomes, followed by self-management support, decision support and clinical information system [10]. With regards to the optional components, majority of the clinics continued with their pre-existing system of chronic disease care.

### The clinical outcomes

The EMPOWER-PAR intervention was proven to be effective in achieving the primary outcome by increasing the proportion of patients who achieved their HbA1c target. Patients in the intervention group were twice more likely to achieve HbA1c target compared to those in the control group (adjusted OR 2.16, 95% CI 1.34–3.50, *P* < 0.002). These findings were similar to the VIDA project, a randomised controlled trial using collaborative learning based on the CCM in Mexico [32]. This study showed that the proportion of patients achieving glycaemic control (HbA1c < 7%) increased from 28 to 39% after 18-month intervention [32]. The interventions in this study were directed at four components of the CCM i.e. self-management support, decision support, delivery system design and clinical information system [32]. Another randomised controlled trial of integrated management of T2DM and depression showed that significantly higher proportion of patients achieved HbA1c < 7% in the intervention group compared to usual care (intervention: 60.9% vs. usual care: 35.7%; *P* < 0.001) [33].

This study also showed that the greatest benefit of intervention was to the poorly controlled patients as the proportion in the ‘improving HbA1c’ category was higher in the intervention (7.3%) compared to the control group (3.2%). This finding is clinically relevant to the Malaysian primary care population as many patients at younger age and those in the early stage of diabetes are being treated in this setting. One of the main clinical indicators for quality management set by the Malaysian T2DM CPG, 5th edition 2015 [34] is to achieve ≥ 30% proportion of T2DM patients in primary care with HbA1c of ≤ 6.5%. Appraisal of evidence by the Malaysian CPG Working Group found strong benefits for reduction of complications at or below this HbA1c level for this group of patients in particular and for the Malaysian population in general [34].

The intervention group showed a reduction in the mean HbA1c while the control group showed an increase instead, (intervention: −0.1% [SE = 0.06] vs. control: 0.2% [SE = 0.09], *P* = 0.003). Although the HbA1c reduction was not clinically impressive, these findings were similar to a randomised controlled trial of a multifaceted diabetes intervention based on the CCM conducted in an underserved community in the United States of America [35]. A modest decline in HbA1c was observed in the CCM group (−0.6%, *P* = 0.008) but not in the provider-education-only group or usual care [35]. Another cluster randomised controlled trial to improve T2DM care in community health centres in the United States showed significant reduction in HbA1c (−0.45%, 95% CI −0.72 to −0.17) after 1–2 years of intervention which incorporated CCM elements [36]. A systematic review on the effectiveness of CCM-oriented diabetes interventions found that CCM interventions were associated with a statistically significant greater mean reduction in HbA1c (−0.46%, 95% CI 0.38 to 0.54; 46 studies) [10].

This study however, did not show significant improvement in the secondary outcome measures i.e. the proportion of patients achieving targets BP, BMI, WC, TC, TG, LDL-c and HDL-c. This is contrary to the findings of a systematic review which found that CCM interventions were associated with significant greater reductions in systolic BP (−2.2 mmHg, 95% CI 0.9 to 3.5; 26 studies), diastolic BP (−1.3 mmHg, 95% CI 0.6 to 2.1; 25 studies) and TC (−0.24 mmol/L, 95% CI 0.06 to 0.41; 17 studies) [10]. The EMPOWER-PAR intervention was proven to be effective in improving the primary outcome i.e. the glycaemic control, but not the secondary outcome measures. However, only the primary outcome was considered in the sample size calculation for this study. Therefore, this study may not be powered to detect the differences in the secondary outcome measures. Another explanation for these findings could be due to the traditional focus of diabetes care in Malaysia towards glycaemic control, while the management of coexisting CV risk factors has been shown to be suboptimal [8]. In EMPOWER-PAR, although utilisation of the Global CV Risk Self-Management Booklet was part of the intervention, its effectiveness in improving the secondary outcome measures has not been demonstrated. When resources were limited, the CDM Team in the intervention clinics may be more focused to improve the HbA1c target, but not the other clinical outcomes. This highlights the need to channel the resources appropriately and to continuously train primary care providers to change the paradigm of diabetes care towards the global CV risk factors approach [37].

### Strengths and limitations of the study

The key strength of EMPOWER-PAR was its pragmatic cluster randomised trial design, which was expected to measure the degree of beneficial effect of the intervention in real life clinical practice. In pragmatic trials, a balance between external validity (generalisability of the results) and internal validity (reliability or accuracy of the results) needs to be achieved [21]. Frequently, cluster randomised trials have a risk of bias due to the allocation of intervention by clusters [38], thus limiting their internal validity. The EMPOWER-PAR reduced the likelihood of bias in allocation by matching the clinics for their geographical locations, staffing and workload. Matching in pairs based on the similarity of the covariates prior to random treatment assignment can greatly improve the efficiency of causal effect estimation [39]. Therefore, when pairing is feasible, clusters should be paired prior to randomisation to minimise bias and to improve efficiency, power and robustness [40]. In this study, the clinics were matched prior to sampling, hence resulting in the comparability of clinics recruited into the study. As randomisation was subsequently done based on pairs of clinics with matched characteristics, this further reduced the risk of bias in cluster allocation. In addition to limiting the risk of bias at the design stage, the analysis of the results took into account the effect of clustering. Baseline covariates between the intervention and control groups were well balanced for almost all covariates suggesting a lack of selection bias during recruitment. The low rates of loss to follow-up (2–3%) minimised selection bias as well. Given its pragmatic trial design and lack of bias, the results of this study may therefore be generalisable to other Malaysian public primary clinics in resource-constrained setting which share similar characteristics.

The EMPOWER-PAR utilised resources which were readily available within the public primary care system in its intervention components. Despite the modest results obtained, this study shows that even without substantial additional resources, developing countries can still effect a change in clinical practice. Findings of this study provide critical supportive information for any developing country with limited resources, as the intervention would probably be inexpensive to replicate. However, cost-effectiveness analyses are required to inform further decision making on the value of the EMPOWER-PAR intervention, and this will be reported in a separate paper.

Another key strength of this study is the PAR approach. In PAR, the researchers attempt to democratise the research process [20]. The iteration of reflection and self-analysis of the intervention, together with the power sharing in the research process are the main characteristics of PAR [20]. In this study, primary care providers in the intervention clinics who were passive players in the beginning became active players as the study progressed. The PAR approach required active participation of the CDM Team from each clinic to design, propose and implement the intervention. The FMS who led the CDM Team in each clinic was also involved in the designing process and ensured that the intervention was delivered as intended. The process of PAR allowed the primary care providers in this study to have increased autonomy to design the intervention plan based on the CCM elements and made the choice of actions within their constraints to improve their patients’ health outcomes. Successful implementation of a complex, multifaceted CCM intervention may depend not only on the provision of appropriate resources and the development of effective systems and processes, but also on the various stakeholders who will interpret and influence the implementation process [41]. Human factors, including the role of healthcare providers and their leaders who can either facilitate or impede successful implementation, should be considered [41]. The PAR approach also allowed collegial environment to develop between facilitators and the CDM Team in this study. This factor may have promoted better reflective practice and could have contributed towards the improved outcomes. In addition to ensuring appropriate resources, successful implementation of CCM interventions would highly depend on whether the intervention is acceptable to both patients and healthcare providers [41]. Primary care providers must be actively involved in the change process to ensure that patients are supported throughout the implementation of CCM interventions.

Limitations of this study include the challenge to ensure implementation fidelity of the EMPOWER-PAR intervention. Monitoring the intervention and ensuring its implementation posed a great challenge to the researchers in this study. It required multiple visits and encounters with primary care providers in the intervention clinics to ensure that the intervention was delivered as intended. Some of the intervention clinics faced constraints such as high staff turnover, high workload and limited consultation time. Despite the constraints, all five clinics were able to optimally adhere to the proposed intervention plan and delivered the obligatory components as intended. Evidence have shown that moderate adherence to a prescribed protocol was more predictive of good intervention outcomes than a perfect level of adherence [42]. This suggests that some level of practitioner flexibility and adaptability is needed to meet local and individual needs when implementing interventions in different populations within different contexts [28]. The optimal implementation fidelity of the EMPOWER-PAR intervention was achieved through tailoring the needs and constraints of each individual clinic. Variations in implementation between each clinic were also found to be minimal and therefore, it is unlikely that this would have influenced the final outcomes.

### Implications for clinical practice, future research and policy change

The EMPOWER-PAR has demonstrated that multifaceted interventions based on the CCM was effective in improving the proportion of T2DM patients achieving HbA1c target in a resource-constrained public primary care setting. Due to its pragmatic design which utilised readily available resources, the results may be generalisable to other primary care clinics in resource-constrained setting which share the same characteristics. However, this study falls short in demonstrating effectiveness in improving the secondary outcomes. This highlights the pressing need to change the paradigm of diabetes care among primary care providers towards the global CV risk factors approach, as there were conclusive evidence that BP, lipid and weight lowering reduced cardiovascular morbidity and mortality among T2DM patients [37].

The primary outcome of this study was set according to the recommendation by the Malaysian T2DM CPG, 4th edition 2009 [27] to avoid confusion among the healthcare providers. The HbA1c target of < 6.5% is quite tight and it is difficult to achieve in real life clinical practice without predisposing patients to hypoglycaemia. This strict target may also be challenged by recent evidence and other international guidelines which recommend target HbA1c of < 7.0% (<53 mmol/mol) [43, 44]. However, the recent Malaysian T2DM CPG, 5th edition 2015 still recommends HbA1c target of ≤ 6.5%, especially for patients with shorter duration of diabetes, no evidence of significant CVD, longer life expectancy and minimal risk of hypoglycaemia [34]. Majority of patients being treated in primary care fit these profiles. In clinical practice, however, HbA1c target should be individualised according to the complexities of individual patient needs to minimise the risk of hypoglycaemia [34].

This study invites further research question whether the intervention and its beneficial effect would be sustainable in the long term. Given the constraints in the Malaysian public primary clinics such as high staff turnover, further research which includes a longer duration of intervention is needed to evaluate the sustainability of the intervention and its effectiveness. Further research which includes public primary care clinics in other parts of Malaysia, which may have different resource constraints, is also needed to provide more robust evidence on the effectiveness of the EMPOWER-PAR intervention.

Policy change and better resource allocations are needed to implement these multifaceted interventions in the Malaysian public primary care setting to ensure its sustainability. There is a need for a holistic understanding among policy makers, healthcare providers and patients, of the complexity of diabetes care in order to instigate change in the management of diabetes in the community [45]. Decision makers need to be able to appraise research evidence judiciously to select cost-effective interventions which could potentially improve outcomes of diabetes care in the community [45]. It is hoped that the evidence from this study will provide a platform to instigate the much needed policy change and resource allocations to support diabetes care in the Malaysian public primary care setting.

## Conclusions

Findings from this pragmatic clinical trial provide objective evidence of the effectiveness of the EMPOWER-PAR intervention in improving the proportion of T2DM patients achieving glycaemic target in real life public primary practice in Selangor and Kuala Lumpur, Malaysia. The results may be generalisable to other Malaysian public primary clinics or other clinics in resource-constrained setting which share the same characteristics. As the intervention utilised readily available resources, it would probably be inexpensive to replicate. However, given the constraints in the Malaysian public primary clinics such as high staff turnover, further research is needed to evaluate whether the intervention and its beneficial effect would be sustainable in the long term. Finally, we hope that the evidence from this study will influence policy change and resource allocations to support management of T2DM in the Malaysian public primary care setting.

## Additional files

Additional file 1: Eligibility assessment of the public primary care clinics, *n* = 34 (PDF 234 kb)
Additional file 2: Definition of outcome categories at 1-year follow-up (PDF 230 kb)

## Abbreviations

BMI: Body mass index
BP: Blood pressure
CCM: Chronic care model
CDM: Chronic disease management
CI: Confidence interval
CPG: Clinical practice guidelines
CRF: Case report form
CV: Cardiovascular
FMS: Family medicine specialist
GEE: Generalised estimating equation
HDL-c: High density lipoprotein-cholesterol
ICC: Intra-cluster correlation coefficients
LDL-c: Low density lipoprotein-cholesterol
MOH: Ministry of Health
NCD-1M: Non-communicable disease – 1 Malaysia
NDR: National Diabetes Registry
OR: Odd ratio
PAR: Participatory action research
QR: Quick references
SE: Standard error
SEL: Selangor
SFQ: Site feasibility questionnaire
T2DM: Type 2 diabetes mellitus
TC: Total cholesterol
TG: Triglycerides
UiTM: Universiti Teknologi MARA
WC: Waist circumference
WHO: World Health Organization
WPKL: Wilayah Persekutuan Kuala Lumpur
