# Supplementary material for: Effectiveness of the EMPOWER-PAR Intervention in Improving Clinical Outcomes of Type 2 Diabetes Mellitus in Primary Care: A Pragmatic Cluster Randomised Controlled Trial
Source: BMC Fam Pract. 2016 Nov 14;17:157. doi: 10.1186/s12875-016-0557-1 (PMC5109682; doi:10.1186/s12875-016-0557-1)
Supplement: Additional file 1: — Eligibility assessment of the public primary care clinics, n = 34 (PDF 234 kb) [file 12875_2016_557_MOESM1_ESM.pdf]

Clinic No.	Number of T2DM patients in the registry (eligible to enroll > 500)	FMS was willing to participate and lead the intervention	Capacity to implement the obligatory component of EMPOWER-PAR intervention			Distance to the central laboratory (in km)	Eligibility to participate
			Delivery System Design: Multi-Disciplinary Team	Self-Management Support: Training & Tool	Decision Support: CPG Training		
1	1800	Yes	Yes	Yes	Yes	55	√
2	550	Yes	Yes	Yes	Yes	39	√
3	2480	Unsure	No	Yes	Yes	50	×
4	2114	Yes	Yes	Yes	Yes	34	√
5	2000	Yes	Yes	Yes	Yes	28	√
6	2060	Yes	Yes	Yes	Yes	34	√
7	850	Unsure	No	No	No	31	×
8	2639	Yes	Yes	Yes	Yes	23	√
9	2400	Unsure	No	No	Yes	12	×
10	1500	No	Yes	Yes	Yes	33	×
11	1500	Yes	Yes	Yes	Yes	35	√
12	3000	Unsure	No	No	Yes	29	×
13	4900	No	Yes	Yes	Yes	20	×
14	2000	Yes	Yes	Yes	Yes	26	√
15	4400	Yes	Yes	Yes	Yes	4	√
16	1878	Yes	Yes	Yes	Yes	50	√
17	1788	Yes	Yes	Yes	Yes	18	√
18	2000	Unsure	No	No	Yes	38	×
19	2000	Yes	Yes	Yes	Yes	20	√
20	4473	Unsure	No	Yes	Yes	46	×
21	1149	Unsure	No	No	No	77	×
22	5521	Yes	Yes	Yes	Yes	33	√
23	1840	Unsure	No	No	No	42	×
24	2500	No	Yes	Yes	Yes	28	×
25	3400	Unsure	No	Yes	Yes	32	×
26	3200	Yes	Yes	Yes	Yes	28	√
27	1800	Unsure	No	Yes	Yes	34	×
28	4000	Yes	Yes	Yes	Yes	30	√
29	2582	Yes	Yes	Yes	Yes	26	√
30	5720	Yes	Yes	Yes	Yes	28	√
31	3100	Yes	Yes	Yes	Yes	55	√
32	2686	Yes	Yes	Yes	Yes	32	√
33	1000	Yes	Yes	Yes	Yes	51	√
34	4000	Unsure	No	Yes	Yes	14	×
