# Supplementary material for: Effectiveness of the EMPOWER-PAR Intervention in Improving Clinical Outcomes of Type 2 Diabetes Mellitus in Primary Care: A Pragmatic Cluster Randomised Controlled Trial
Source: BMC Fam Pract. 2016 Nov 14;17:157. doi: 10.1186/s12875-016-0557-1 (PMC5109682; doi:10.1186/s12875-016-0557-1)
Supplement: Additional file 2: — Definition of outcome categories at 1-year follow-up (PDF 230 kb) [file 12875_2016_557_MOESM2_ESM.pdf]

Categories of Outcome	Baseline	1-year follow-up
Definitions for HbA1c (%)		
Deteriorating	< 6.5	≥ 6.5
Poor, No change	≥ 6.5	≥ 6.5
Good, No change	< 6.5	< 6.5
Improving	≥ 6.5	< 6.5
Definitions for BP (mm/Hg)		
Deteriorating	≤ 130/80	> 130/80
Poor, No change	> 130/80	> 130/80
Good, No change	≤ 130/80	≤ 130/80
Improving	> 130/80	≤ 130/80
Definitions for BMI (kg/m^2)		
Deteriorating	< 23	≥ 23
Poor, No change	≥ 23	≥ 23
Good, No change	< 23	< 23
Improving	≥ 23	< 23
Definitions for WC (cm)		
Deteriorating	M < 90<br>F < 80	M ≥ 90<br>F ≥ 80
Poor, No change	M ≥ 90<br>F ≥ 80	M ≥ 90<br>F ≥ 80
Good, No change	M < 90<br>F < 80	M < 90<br>F < 80
Improving	M ≥ 90<br>F ≥ 80	M < 90<br>F < 80
Definitions for TC (mmol/L)		
Deteriorating	≤ 4.5	> 4.5
Poor, No change	> 4.5	> 4.5
Good, No change	≤ 4.5	≤ 4.5
Improving	> 4.5	≤ 4.5
Definitions for TG (mmol/L)		
Deteriorating	≤ 1.7	> 1.7
Poor, No change	> 1.7	> 1.7

Good, No change	≤ 1.7	≤ 1.7
Improving	> 1.7	≤ 1.7
Definitions for LDL-c (mmol/L)		
Deteriorating	≤ 2.6	> 2.6
Poor, No change	> 2.6	> 2.6
Good, No change	≤ 2.6	≤ 2.6
Improving	> 2.6	≤ 2.6
Definitions for HDL-c (mmol/L)		
Deteriorating	≥ 1.1	< 1.1
Poor, No change	< 1.1	< 1.1
Good, No change	≥ 1.1	≥ 1.1
Improving	< 1.1	≥ 1.1
